# The compensatory potential of increased immigration following intensive American mink population control is diluted by male-biased dispersal

**DOI:** 10.1007/s10530-016-1199-x

**Published:** 2016-07-01

**Authors:** M. K. Oliver, S. B. Piertney, A. Zalewski, Y. Melero, X. Lambin

**Affiliations:** 1 School of Biological Sciences, University of Aberdeen, Aberdeen, AB24 2TZ UK; 2 Mammal Research Institute, Polish Academy of Science, 17-230 Białowieża, Poland; 3 Present Address: Centre de Recerca Ecològica i Aplicacions Forestals (CREAF), Universitat Autònoma de Barcelona, Bellaterra, Barcelona, Spain

**Keywords:** Mink, Control, Compensation, Immigration, Dispersal, Hotspots

## Abstract

Attempts to mitigate the impact of invasive species on native ecosystems increasingly target large land masses where control, rather than eradication, is the management objective. Depressing numbers of invasive species to a level where their impact on native biodiversity is tolerable requires overcoming the impact of compensatory immigration from non-controlled portions of the landscape. Because of the expected scale-dependency of dispersal, the overall size of invasive species management areas relative to the dispersal ability of the controlled species will determine the size of any effectively conserved core area unaffected by immigration from surrounding areas. However, when dispersal is male-biased, as in many mammalian invasive carnivores, males may be overrepresented amongst immigrants, reducing the potential growth rate of invasive species populations in re-invaded areas. Using data collected from a project that gradually imposed spatially comprehensive control on invasive American mink (*Neovison vison*) over a 10,000 km^2^ area of NE Scotland, we show that mink captures were reduced to almost zero in 3 years, whilst there was a threefold increase in the proportion of male immigrants. Dispersal was often long distance and linking adjacent river catchments, asymptoting at 38 and 31 km for males and females respectively. Breeding and dispersal were spatially heterogeneous, with 40 % of river sections accounting for most captures of juvenile (85 %), adult female (65 %) and immigrant (57 %) mink. Concentrating control effort on such areas, so as to turn them into “attractive dispersal sinks” could make a disproportionate contribution to the management of recurrent re-invasion of mainland invasive species management areas.

## Introduction

The feasibility of control or eradication is a central question for invasive species management efforts aiming to limit the impacts of invasive non native species on native biodiversity (Saunders and Norton 2001). Understanding the factors and processes that affect the outcome of invasive species control strategies is therefore fundamentally relevant to the success of ISM initiatives and *vis a vis* the conservation of biodiversity. The main contemporary focus of mammalian invasives management efforts has been concentrated on islands, where a number of well documented successes have been achieved (e.g. Courchamp et al. 1999; Barun et al. 2011; Kessler 2011). Glen et al. (2013) convincingly argued that management should embrace a landscape planning approach so as to maximise conservation benefits, and indeed the perspective of management has broadened with an appreciation of the necessity to address the effect of species invasions at larger spatial scales, where target areas may be nested within large land masses. Some such large-scale projects take a “Mainland Island” approach and seek to create a contiguous area in which immigration by invasive species can be limited. Boundaries may be defined by capitalising on semi-permeable habitat barriers (e.g. Zalewski et al. 2009; Fraser et al. 2013), but buffer areas with a degree of invasive species control have also been used effectively (Thomson et al. 2000; Kinnear et al. 2010). Because non-ecological factors such as availability of financial resources and the protection designation of land masses often define management areas (e.g. Nordström et al. 2003), protected areas may be surrounded by, or interspersed with, areas where invasive species are left unmanaged.

Controlling invasive species in portions of landscapes will inherently be countered by ongoing reinvasion from adjacent uncontrolled areas by invasive species that, by virtue of their invasiveness, have a high dispersal ability. Thus, rather than achieving eradication, the management objective in landscape scale management is to depress invasive species numbers to near-zero density, or to a level where their impact on native fauna in core conservation areas is tolerable [e.g. stoats on Secretary Island, NZ (McMurtrie et al. 2011), and red foxes in Australia (Moseby and Hill 2011). Given that management must continue in perpetuity in such circumstances, it is essential to optimise the conservation return from investment by drawing upon and improving our understanding of such managed systems. Objectives are twofold: firstly, effectively reducing the target invasive species population(s) in focal core areas; and second, maintaining low numbers by minimising reproduction and compensatory immigration. The former may be achieved by optimising the effectiveness of the removal process. For example, using traditional knowledge of the locations of red fox dens to target spring culling has proven to be the most effective way of reducing densities of this widely controlled predator because there is little scope for reinvasion within the breeding season (Heydon and Reynolds 2000). The latter objective requires an understanding of the drivers of dispersal along gradients of density.

In addition to compensatory natality or mortality, compensatory immigration—an increase in immigration rates following a reduction in local density through control efforts—often occurs into areas following mortality through natural or anthropogenic factors (Gervasi et al. 2015). This can rapidly restore population sizes toward pre-disturbance levels (Turgeon and Kramer 2012). Such compensatory fluxes in dispersal can also limit the effectiveness of control efforts by extending the persistence of controlled populations (Lieury et al. 2015). Unlike compensatory natality, or mortality that involves in situ survivors and is therefore not strongly affected by scale, compensatory immigration is affected by factors both within, and external to, the focal control area. Where immigration compensates for culling, only a core area may experience reduced abundance of the target species. Conversely, in some circumstance the impact of culling may extend beyond the controlled area, creating a “halo effect” through its influence on individuals (Glen et al. 2013). Because of the expected scale-dependency of compensatory immigration, small management areas are expected to have a smaller core area unaffected by immigrants from surrounding areas if the spatial scale of control is not sufficiently large relative to the scale of dispersal of the focal controlled species.

Key factors that are predicted to dictate the dynamics of compensatory immigration include: the abundance, and gradients in the abundance of potential immigrants with distance from source areas; the ability of those immigrants to reach and detect low density optimal areas; and the degree of heterogeneity in the productivity and spatial structure of habitat, which, in combination with density, define the quality of available settlement areas (Fretwell and Lucas 1969; Efford et al. 2000; Delibes et al. 2001a, b). Compensatory immigration rates are expected to increase where immigrants are able to detect and select optimal habitat patches, which in turn depends on the ability of transient individuals to remain in the dispersal phase and gather information on their environment for protracted periods of time. When information on habitat quality is available to dispersers, the highest quality depleted habitats are expected to be recolonised first, and then for compensation through dispersal to be maximally effective.

Amongst invasive species, several medium size generalist carnivores (e.g. red fox, American mink, mongoose, ferrets, stoats and feral cats) have colonised large new areas where they have established high density populations (Park 2004). In their new invasive ranges these species typically experience low levels of predation, and with high inherent mobility and generally broad diets, are able to disperse over large distances, acquiring information on the variation in the quality of the environment hence to make “informed dispersal decisions” and thus make dispersal more demographically effective in compensating for culling (Santini et al. 2014). Dispersal is male-biased in those medium size invasive generalist carnivores, as it is in most mammals, and primarily takes place prior to breeding. When dispersal is male-biased, males may be overrepresented during compensatory immigration, resulting in a male-biased population with a lower potential growth rate in re-invaded areas, though the extent of this will vary between species.

Obtaining adequate information on how dispersal patterns combine to predict recolonisation pressure for highly mobile, low density invasive predators is exceptionally challenging. As a result, there is a knowledge gap around how such controlled populations will numerically respond to control efforts given the spatial scale of control relative to the spatial scale of dispersal processes, and management actions are thus often undertaken from a relatively uninformed baseline (Cook et al. 2010). Here we use data collected from a large scale invasive species control project to evaluate the impact of immigration on the success of American mink (*Neovison vison*) control in NE Scotland, investigating heterogeneity in productivity, the extent of dispersal and landscape connectivity, and changes in sex ratio and immigration following population culling.

### Study system and questions

This study is framed around the Cairngorms Water Vole Conservation Project (CWVCP), which was initially conceived to protect remnant populations of water voles (*Arvicola amphibius*) in the headwaters of the Cairngorm Mountains in northeast Scotland, UK (Bryce et al. 2011). The project was initiated following a catastrophic UK-wide decline in water vole distribution, which has been attributed to predation by the introduced and invasive American mink, and to protect the large continuous metapopulation networks of this species that persisted in the area (Aars et al. 2001).

**Fig. 1:**
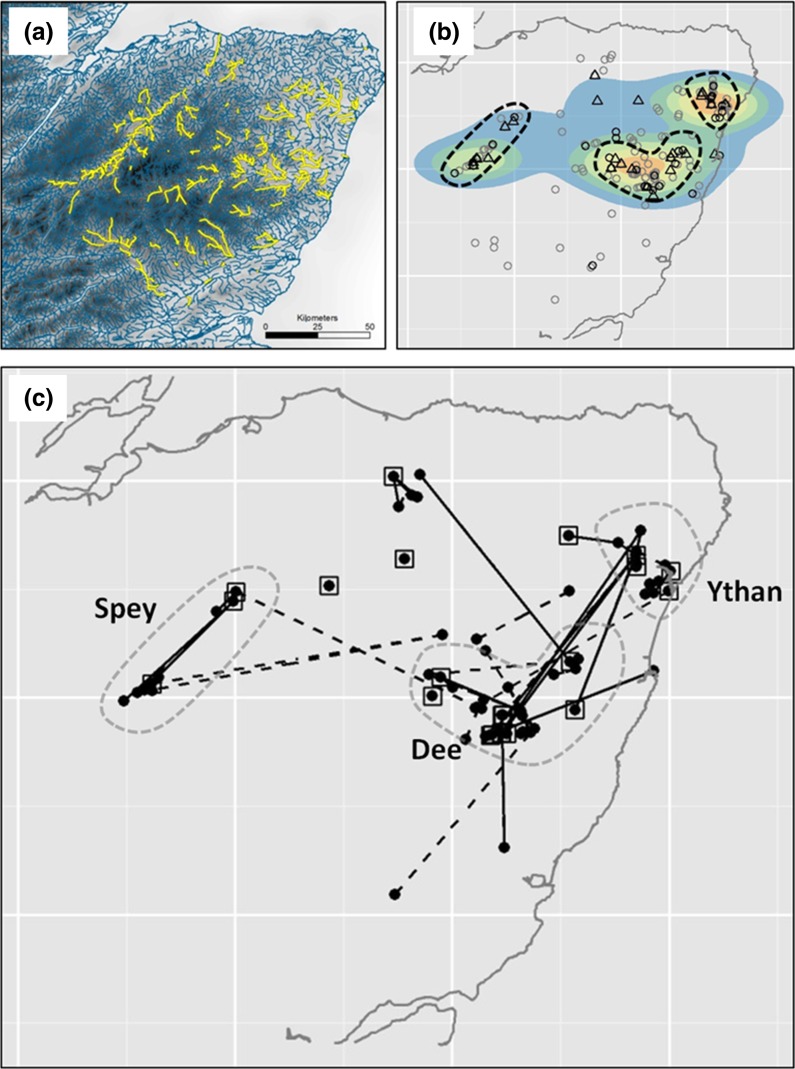
Maps of the study area showing: **a** Waterways covered by the mink raft network. Variation in landscape tone illustrates altitude, with darker areas representing uplands, the darkest being mountains ≥1000 m, and palest grey the North Sea. **b** Hotspots of mink productivity with the capture locations of adult female (*black triangles*), juvenile (*black circles*), and adult male and non-settled subadult mink (*grey circles*). *Colours* are generated by heat plots with the relative density of adult female and juvenile mink represented from high (*red*) to low (*blue*). **c** Dispersal movements inferred through pedigree reconstruction. *Squares* are the sources of movements (including no movement) inferred from a young (pre-dispersal) sibling. *Black dots* are the capture locations of inferred dispersers. Movements from an inferred source are depicted by a *solid line*, whereas connections between individuals from the same litter, but where no source is known, are depicted by straight *broken lines*. The *hatched line* polygons show areas identified as hotspots of both breeding and dispersal nodes. The 3 river catchment areas that encompass the hotspot areas are labelled on the *bottom panel*

The project area gradually expanded to eventually encompass *ca.* 10,000 km^2^ of northeast Scotland by 2009, incorporating seven major river catchments distributed on both sides of the Cairngorm Mountains (Fig. 1a). The landscape is highly heterogeneous and includes relatively unproductive upland, moorland and mountain habitats that are bisected by river valleys, which in turn drain into productive, lowland agricultural areas. Land management interests, namely grouse shooting, salmon fishing, conservation and farming vary between river catchments. This heterogeneity and associated variation in the existence and organisational strength of interest groups (e.g. land estates; rivers and salmon fishery trusts; and organised conservation volunteers) contributed to variation in the timing of inception of mink control. Thus the project expansion from the headwaters towards the lowland coastal plain was non-systematic and left, for a time, a patchwork of areas varying from recently to long-established mink control. Throughout, there was scope for mink control in a given catchment being negated by immigration from both adjoining and/or further afield catchments, where control was less advanced.

At the outset of the conservation project our understanding of potentially important facets of mink compensatory responses to culling was limited. However, mink had been reported through most of the project area (NBN 2009) and there existed a useful body of literature on mink demography, spatial ecology, habitat and dietary preferences (e.g. Yamaguchi et al. 2003; Yamaguchi and Macdonald 2003; Bonesi and Harrington 2006; Bonesi et al. 2007), though little for upland areas, or across habitat gradients.

In this paper we aim to characterise: (1) the spatial scale of mink dispersal and landscape connectivity; (2) the degree of spatial heterogeneity in mink productivity; and (3) the change in the frequency of immigrants following mink control. These relate respectively to three applied questions: (1) what is the appropriate spatial scale for control efforts to achieve project goals? (2) Should we prioritise resources and effort towards particular areas? (3) What is the risk that immigration will overcome control efforts?

## Methods

Mink carcasses were collected between 2003 and 2009 from an area of North East Scotland incorporating seven major river catchments [the rivers Dee (57.1171°N, 2.1141°W), Deveron (57.4546°N, 2.7942°W), Don (57.1250°N, 3.2734°W), North Esk (56.7517°N, 2.4317°W), South Esk (56.8987°N, 3.2542°W), Tay (56.3917°N, 3.4251°W) and Ythan (57.4319°N, 2.2463°W)], initially focussing on subcatchments of the Dee, Don and Ythan and expanding to a coverage area of *ca*. 10,000 km^2^ (1303 km of waterways) by 2009. A total of 301 mink (88 mink from 2003- May 2006, and 213 from June 2006 to 2009) were captured using a detect-then-trap approach, utilising 932 Game Conservancy Trust mink rafts (Reynolds et al. 2004) run by CWVCP project officers and volunteers. Captured mink were humanely despatched and then kept in freezers prior to laboratory dissection, during which sex was determined, muscle tissue was removed for DNA analysis and canine teeth were removed for ageing. Mink age was determined in two stages. Firstly, canines were X-rayed and two age classes, adults and juveniles, were defined, according to the pulp proportion in the teeth (Drusini et al. 1991). Next, the ages of adults were precisely determined by sectioning and staining teeth to show the cementum lines, performed at Matson’s lab (Montana, USA).

### Capture rates and spatial variation in productivity

Changes in the number of mink captures per km of waterway (assuming uniform detection rates, trapping attempts and success) following population control were estimated by combining mink capture data with the areas with mink raft coverage and effort (years of control) mapped on GIS (ArcGIS 9.3.1 by Esri). The mink raft (a mink-detection device that is anchored to the edge of a waterway consisting of a 120 cm × 60 cm floating platform housing a clay paw print detection pad within a tunnel) network was used to delineate areas connected by mink rafts (using a 500 m buffer either side of a waterway), and an effort matrix was superimposed upon the landscape. Following Bryce et al. (2011), effort was calculated from the time that a river subcatchment was fully covered by mink rafts (or where other appropriate forms of vigilance were in place e.g. active mink trapping by gamekeepers) according to the expert knowledge of the local mink control officer. The standard procedure was for full coverage of river subcatchments to be achieved in <6 months of the first raft deployment. An effort value of 0 was used prior to the river section being fully covered, whereas a value of 1 was used within a year following the time at which the section became fully covered. Thus, the association between calendar year and years of control effort varied in different areas. To assess broad scale spatial variation in mink captures per km, the raft network coverage was divided into a total of 14 sections (mean length = 91 km, median = 103 km, range = 24–152 km, measured on a 1:25,000 scale GIS layer). Each section was a contiguous part of the waterway network with the same history of control effort, and there were between 1–3 sections per major river catchment. The number of mink captures was then calculated for each river section and year of control. To investigate the potential confounding effect of dispersal on mink control, mink captures were additionally separated into two classes: ‘settled’ (all adult females as well as adult males captured outside of the rut period of February and March) and ‘dispersing’ (all subadult mink captured in September–December, and adult males captured in February and March). Note that this subjective discrimination of mink according to life stage is different from the categorisation based on genetic kinship inference used later in this paper. Changes in the number of mink captured following increasing years of control were modelled using a generalised linear mixed model (GLMM) with Poisson errors, and with river section length (the coefficient therefore corresponding to mink captured per km of waterway) and year of control as explanatory variables. River section was included as a random factor to account for non-independence in repeat measurements of mink abundance. All GLMMs were performed using the GLMER function from the lme4 package in R. To illustrate variation in spatial patterns of mink productivity and potential hotspots, the capture locations of adult female (>9 months old) and juvenile mink (<5 months old) were plotted on the landscape. To visually highlight aggregation, a heat map was used (utilising the ggplot and stat_density2d functions of the ggplot2 package in R), where the gradient is calculated using a 2-dimensional kernel density estimate, based on bivariate normal distributions; the density at a point is scaled so that the integral of density over all x and y = 1.

### DNA extraction and genotyping

Molecular analyses were used to address questions concerning mink dispersal, landscape connectivity and immigration. A sample of muscle tissue was removed from a mink carcass during dissection. DNA was extracted using DNeasy according to the manufacturer’s protocol. For all mink, genotyping was performed at 12 microsatellite loci developed for mustelids: Mer009, Mer022, Mer041, Mvi054, Mvi057, Mvi232, Mvi111, Mvi1321, Mvi1381, Mvi1843, Mvis022, Mvis072 (O’Connell et al. 1996; Fleming et al. 1999; Vincent et al. 2003). The 213 mink captured between June 2006 and 2009 were genotyped at an additional three loci: Mvi4001, Mvi4031, Mvi4058 (Anistoroaei et al. 2006). Polymerase chain reaction (PCR) amplifications were performed in a total volume of 10 μL using an MJ Research PTC-100 thermal cycler. Each reaction mix contained approximately 20 ng of template DNA, 2.5 mm MgCl2, 75 mm Tris-HCl (pH 9.0), 20 mm (NH4)2SO4, 0.01 % (v/v) Tween 20, 0.2 mm of each nucleotide, 5 pmol of each primer (only for Mvi232, 2.5 pmol) (forward primer end-labelled with either HEX, NED or 6-FAM fluorescent dyes) and 0.5 U *Taq* polymerase (Bioline Ltd). The PCR profiles for all loci except Mvi054 followed a ‘touchdown’ procedure (Don et al. 1991), whereby after an initial denaturation step of 2 min at 92 °C, 20 cycles of PCR were performed, each cycle consisting of 15-s denaturation at 90 °C, and 15 s of annealing starting at 60 °C and dropping by 0.5° per cycle. A further 18 cycles were then performed with 15-s denaturation at 90 °C and 15-s annealing at 50 °C. No extension steps were included in the programme, except for a 1-min period at 72 °C following the final annealing step. The PCR profile for Mvi054 included an initial denaturation at 94 °C for 1 min 20 s, then 36 cycles of 30-s denaturation at 94 °C and 30-s annealing at 50 °C, with a final extension step of 72 °C for 5 min. Alleles were resolved by electrophoresis on an Applied Biosystems 3730 automated DNA sequencer. Negative extraction and PCR controls were included throughout. Prior to pedigree and kinship analyses, Micro-Checker version 2.2.3 (van Oosterhout et al. 2004) was used to test for stuttering, large allele dropout, and the presence of null alleles. No evidence of stuttering or large allele dropout was detected. The potential presence of null alleles was suggested for four loci (Mvi111, Mer009, Mvi1321, Mvi4001), though the predicted frequencies were low (0.032–0.044).

### Pedigree-based analysis of dispersal and connectivity

Dispersal movements were inferred from the locations of litter mates, which were determined through pedigree analysis using COLONY 2.0 (Jones and Wang 2010). Based on age data, mink were separated into groups of candidate fathers (>8 months old; a male may father offspring, but die prior to their birth) and mothers (>1 year old), or offspring, for each generation (Table 1). Sibships and parentage were then simultaneously assigned using maximum likelihood. This process includes inferring the most likely genotypes of unsampled parents to construct the pedigree. Female American mink produce one litter per year, typically born around April and May (Dunstone 1993). Following tooth structure-based age determination, all individuals were assigned as potential mothers, fathers and offspring for each generation (year of birth). We took a conservative approach to mitigate for uncertainty associated with conducting a pedigree-based analysis on a wild population with partial sampling of individuals and the genome, and uncertainty around the levels of polygamy and inbreeding, two factors known to influence the reliability of pedigree analysis (Wang 2014). The information was intended to determine dispersal movements, and our priority was therefore to minimise error at the cost of reduced data. We varied the COLONY input parameters to perform analyses for both monogamous and polygamous mating systems and also allowed inbreeding to account for population structure. Although mink are known to be polygamous (Yamaguchi et al. 2004), this creates a far more complex problem of pedigree elucidation. We selected the most stringent likelihood settings for COLONY runs, and only considered assignments with probabilities ≥0.8. We then only retained mother-offspring pairs that were assigned from different runs with both mating systems, and sibships that were assigned as full-sibs under monogamy and either full-sibs, or maternal half-sibs, under polygamy. Father-offspring (n = 45 & 55 for monogamy and polygamy, respectively) and paternal half-sibs (n = 271) were not used, as adult male mink are known to range far during mate-searching (5–20 km, Dunstone 1993) or may disperse in the second or third years of their lives, and are therefore of little use in defining the source of dispersal movements based on offspring captures months later. Dispersal was inferred by combining assignment, age, and location data.

**Table 1:** Summary of the numbers of individual mink used as candidates; the number of siblings and mothers assigned to litters (2 or more first order related individuals) from pedigree analysis under monogamous or polygamous mating systems, and the number of individuals that were consistently assigned across both mating systems

Year	Candidates			Monogamy		Polygamy		Consistent	
	Offspring	Fathers	Mothers	Siblings	Mothers	Siblings	Mothers	Siblings	Mothers
2003	34	14	1	15	1	20	1	9	1
2004	17	20	6	8	0	14	1	8	0
2005	22	15	8	11	1	20	2	11	1
2006	29	15	9	19	2	26	3	10	2
2007	85	22	18	65	6	52	5	35	3
2008	90	26	14	61	6	68	6	28	4
Total	277	112	56	179	16	200	18	101	11

Under monogamy, siblings are full-siblings, whereas under polygamy siblings can be full-siblings or maternal half-siblings. The total numbers of candidates exceeds the number of individuals sampled, as the same individual may be present as a candidate offspring or parent in different generations

Following pedigree reconstruction it was shown that individuals from the same litter were captured in relatively close proximity (1st quartile = 0.4 km, median = 1.4 km, 3rd quartile = 4.5 km, n = 34) up to mid-September. As such, the youngest of any member of a litter captured earlier than the 15th of Sept in the year of birth was used as a putative source location for the litter. Where no such individual was available, dispersal distances were estimated from the mean (centroid) coordinates of the litter. These centroid coordinates were used in the estimation of the dispersal kernel, but we did not deem them precise enough to infer specific connections between different river sections and catchments. Dispersal distance was modelled separately for either sex against time (since birth of the litter, assumed to be 1st of May) using an asymptotic regression function (SSasympOff) in R with three parameters: (1) an offset (age in days when dispersal distance = 0); (2) the natural logarithm of the rate constant; and (3) an asymptote. Mink that were older than 250 days (8 months) and that had moved <4 km (based on the ‘spread’ of litter mates captured in the summer months) were removed from this part of the analyses as they were considered to reflect a separate tactic of philopatric settlement that could confound characterisation of dispersal rates, as discussed below.

### Immigration, age structure and sex ratio

Since changes in sex ratio can affect per capita population growth rate, a Chi squared test was used to test whether counts of males and females changed following year of control as a result of sex-biased dispersal. To investigate changes in levels of immigration following population control, a group of conspecifics (individuals that were born prior to and dying after the birth of the focal individual, or that were born prior to, and living beyond, the death of the focal individual) representing potential kin was established for each individual. If individuals removed in the first year of control are replaced by immigrants, then we would predict that individuals captured in year 2 are more likely to have fewer closely related kin in their vicinity. A kin group was defined for each individual captured in areas in the first (n = 155) and second (n = 55) years of full project control (only three individuals were captured in areas with longer histories of control) using Kinship (Queller and Goodnight 2008). Pairwise values (10,000 replicates) of the Kinship coefficient, *r*, were generated using simulations of different genealogical relationships based on the allele frequencies of the total dataset. Comparisons of the simulated distributions of *r* for parent-offspring, full-siblings, half-siblings and non-related individuals suggested that an optimal value of *r* = 0.38 would retain a large proportion of first order relatives (87 % of parent-offspring; 79 % of full-siblings), whilst only including 26 % of half-siblings and 3 % of non-related pairs. The number of conspecifics (all individuals with overlapping life spans) and kin (*r* ≥ 0.38) were recorded within a radius of 10 km (314 km^2^) of the capture location of each individual.

A GLMM with Poisson errors and river section (to account for non-independence) and observation [to account for overdispersion (Harrison 2014)] as random factors was used to examine whether individuals captured in the second year of full control had fewer kin (i.e. were more likely to be immigrants) than those captured in the first year of control. Mink captured prior to the first year of full control were not considered, so as to avoid bias that may be associated with variation in sampling error; monitoring efforts were effectively the same for the areas considered in the 1st and 2nd year of full control, but were patchy and non-systematic in some areas prior to inception of full control. It was necessary to control for the effect of variation in the sampling of potential kin, so the model was defined as the number of kin per conspecific (relative or non-relative) sampled within a 10 km radius. This radius was deemed sufficiently large to adequately incorporate multiple mink territories (Bonesi et al. 2007), whilst limiting dilution of effect sizes through over-scaling on the *x* axis. A second GLMM with binomial errors and the same covariates and random effects was used to test whether the probability of being assigned as a male immigrant (i.e. being male and having no kin within a 10 km radius, as opposed to being male and having kin, or being female) varied between the first and second years of control.

## Results

### Capture rates and spatial variation in productivity

**Fig. 2:**
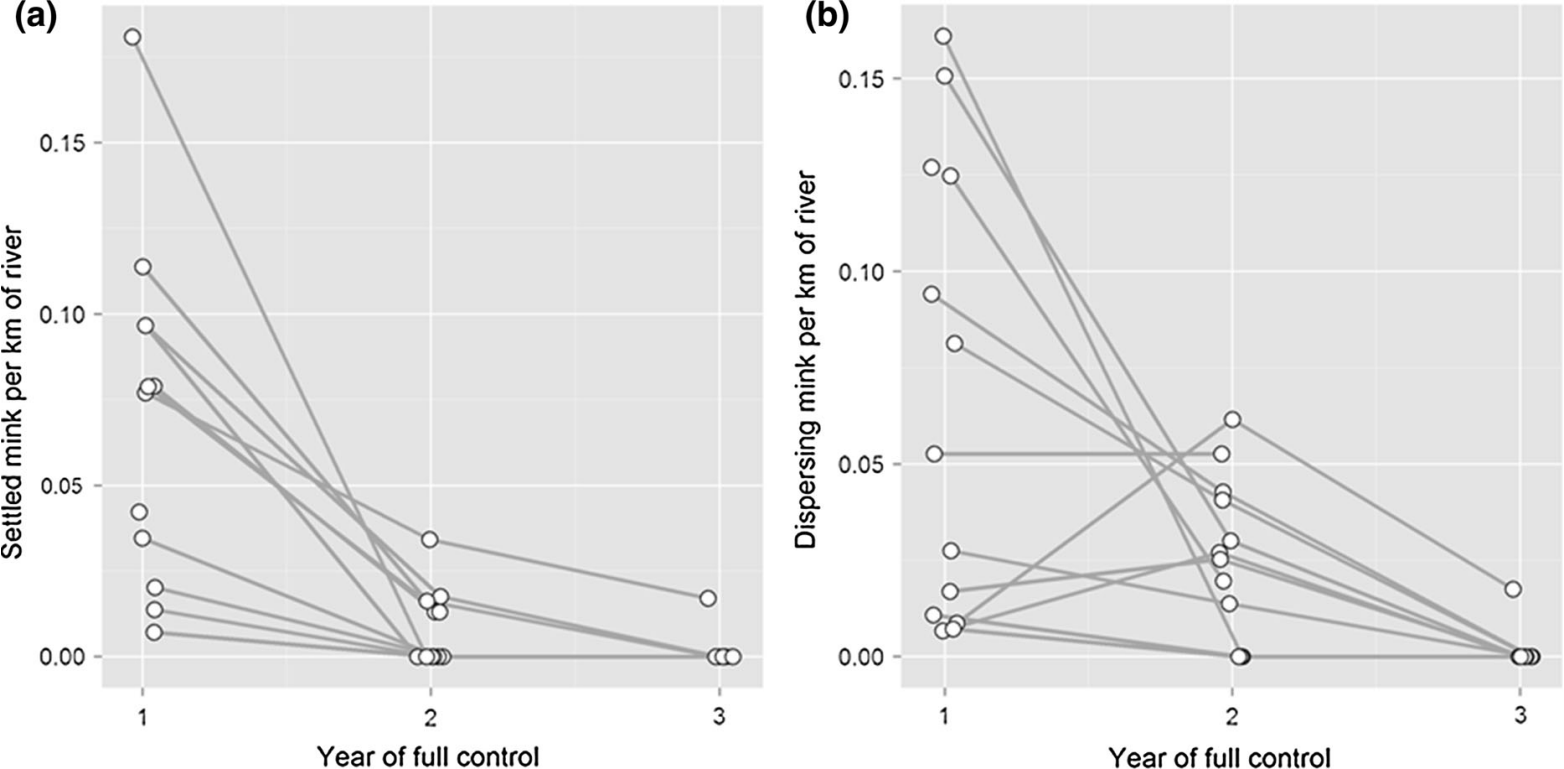
Changes in the number of: **a** ‘settled’ and **b** ‘dispersing’ mink per km of waterway in river sections with increasing years of mink control and comprehensive coverage under the conservation project mink raft network. Points have been offset on the *x* axis for clarity

Across river sections the number of mink captured per km of waterway showed a clear decrease with increasing years of mink control, from an average of 0.16 to 0.06 to 0.01 for river sections in the first, second and third years of control respectively, although there was variability in the trend, with increases in some river sections between the first and second years of control. Separately analysing mink from ‘settled’ and ‘dispersing’ life stages showed that the variability between river sections in declining trend was due to mink that were in dispersing life stages (rut males searching for female mates, or dispersing subadults putatively searching for a territory) (Fig. 2). Modelling mink captures against year of control, whilst controlling for section length as a covariate and section ID as a random effect, showed that mink capture rate declined significantly year on year of control for both settled *p* < 0.0001 for all between-year contrasts, n = 45, Fig. 2a) and dispersing mink (*p* < 0.002 for all between-year contrasts, n = 45, Fig. 2b). However, the decline in mink capture rate was much stronger for settled mink, where an average of 0.7 mink were captured per river Sect. (0.007 settled mink/km of river) compared to 2.2 dispersing mink per river Sect. (0.024 dispersing mink/km of river) in the second year of control, despite very similar capture rates and densities for the two classes of mink in the first year of control. Captures of settled mink declined in all river sections with increasing years of control, excluding two river sections where no territorial mink were ever caught (Fig. 2a). In contrast, capture rates of dispersing mink were more variable, with increases or constancy in three and one sections respectively, while capture rates decreased in the others, again excluding one section where no settled mink were ever caught (Fig. 2b).

Settled mink were not distributed uniformly between river sections in the first year of mink control, but were instead highly aggregated. Sixty one of 68 (90 %) were caught in only seven river sections accounting for 50 % (575 km) of the length of waterways, a significant deviation relative to expectation under a uniform distribution (χ^2^ = 42.8, *df* = 1, *p* < 0.01). We refer to these areas as ‘hotspots’. Variation in initial densities was not attributable to a broad scale temporal variation in mink density, as all results were robust to including initial year of coverage in the models.

### Pedigree analysis, mink dispersal and connectedness of river catchments

One hundred and ninety five and 218 individuals (mothers and offspring) were assigned to a litter based upon a monogamous or polygamous mating system, respectively. One hundred and twelve of these were consistent, irrespective of the assumed mating system and were hence conservatively retained. These 112 individuals (including mothers) formed 47 litters of mean size 2.4 maternal siblings, with a biologically realistic range of 2–6. Only 11 of 47 litters (23 %) were assigned a mother from the candidate parents, meaning that the mothers of 36 litters were either not sampled, or could not be confidently distinguished amongst genetically similar candidates. The indeterminacy of the pedigree reconstruction process was further highlighted by 40 (71 %) and 38 (68 %) of 56 candidate mothers having no offspring assigned at a probability of ≥0.8 under monogamy and polygamy, respectively. Ninety eight (35 %) and 77 (28 %) of 277 candidate offspring had no mother assigned using either mating assumption, an increase of 21 under the more lax assignment criterion (Table 1). Here, true mothers could have avoided capture within the trapped area, failed to be confidently discriminated within the sample, or alternatively, the candidate offspring could have immigrated from areas beyond the trapped river sections.

**Fig. 3:**
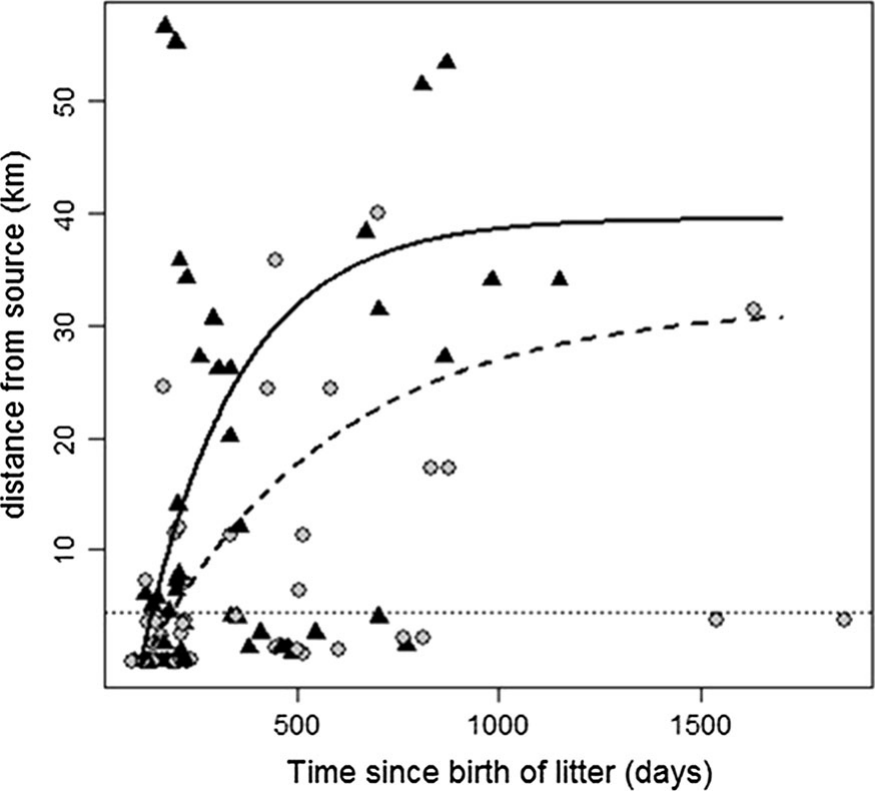
Dispersal kernels for female (*grey circles*, *hatched line*) and male (*black triangles*, *solid line*) mink, modelled using a 3 parameter model. Individuals older than 250 days that had moved <4 km were categorised as non-dispersers (*dotted line*) and were not included in the estimation of dispersal kernels

Most mink were caught away from the inferred natal location, i.e. started dispersing from around mid—September in the year of birth, with males dispersing faster (*c.*25 km in 6 months, estimated asymptote = 39.5 km, SE 7.1 km, *p* < 0.001) and further than females (*c.*7.5 km in 6 months, estimated asymptote = 31.8 km, SE 7.6 km, *p* < 0.001) (Fig. 3). Sixteen percent of adult mink (>250 days (8 months) old: 9 males, 9 females) appeared not to have dispersed from around the natal area, having moved <4 km at the time of capture (individuals below the hatched horizontal line, Fig. 3).

Individuals from 15 of the 47 inferred litters (32 %) were captured in different river catchments, implying overland dispersal (Fig. 1c). Fourteen of these were litters to which only two individuals had been assigned, with the other being one litter to which three individuals had been assigned. For 12 of the 15 cases where littermates were captured in different catchments, these were not adjacent, but were instead interposed by either other major river catchments, or moorland ridges (see Fig. 1c in conjunction with 1a).

Low sample sizes precluded any formal significance testing, but notwithstanding, movement patterns suggest lowland habitats are more permeable to mink movement than moorland habitat. Indeed 10 of 15 inferred dispersal events between catchments linked lowland river sections (lighter background in Fig. 1a; see 1c for movements). The 5 movements between areas separated by moorland (dark background in Fig. 1a; see 1c for movements) could conceivably have circumvented moorland habitat.

The areas identified as hotspots of productivity also received a disproportionately high number of identified immigrants, with 45 of 65 individuals from 19 litters where a source of dispersal was identified, and that were of dispersal age (>5 months) being captured here, and 57 % of dispersal movements (>4 km) ending in these areas, *despite* them only representing 40 % of the total waterways, a highly significant difference from null expectation (χ^2^ = 11.1, *df* = 1, *p* < 0.001).

### Changes in sex ratio and immigration following population control

**Fig. 4:**
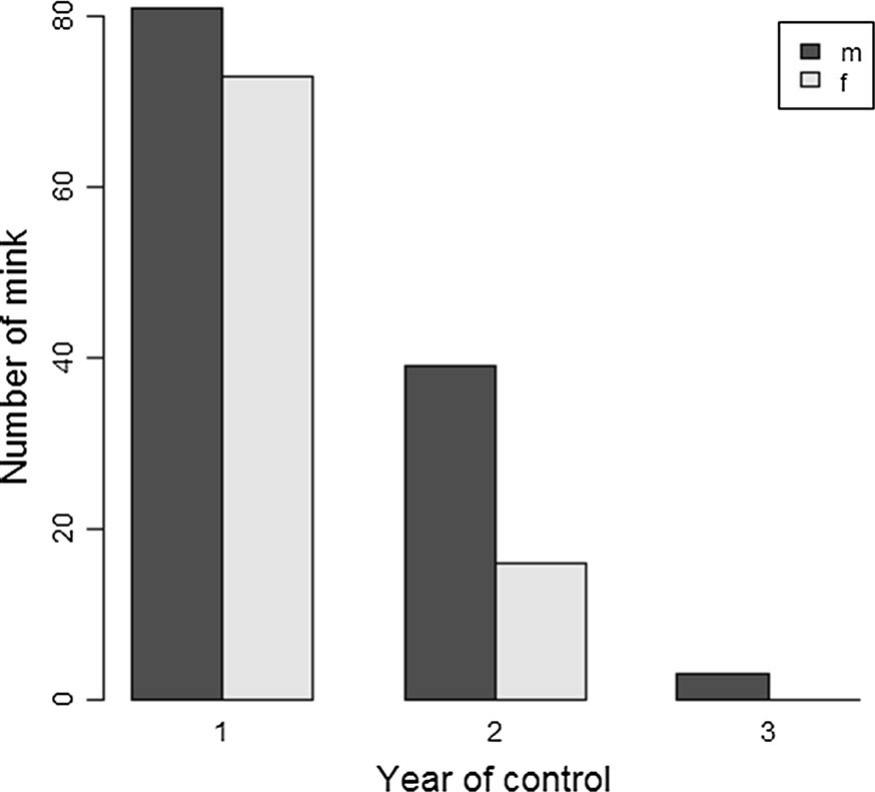
Changes in the sex ratio of mink captured in areas with different years of comprehensive raft coverage and mink control

There was a substantial and significant increase in the ratio of male to female mink with increasing year of control (*X* ^2^ = 7.78, *df* = 2, *p* = 0.02), from a sex ratio that was close to parity (53 % males) in areas in the first year of full control to almost two and half times as many males (71 %) than females being captured in areas in the second year of full control (Fig. 4). All of the three individuals captured in river sections in a third year of control were subadult males (and also classed as likely to be immigrants from kinship analysis).

**Fig. 5:**
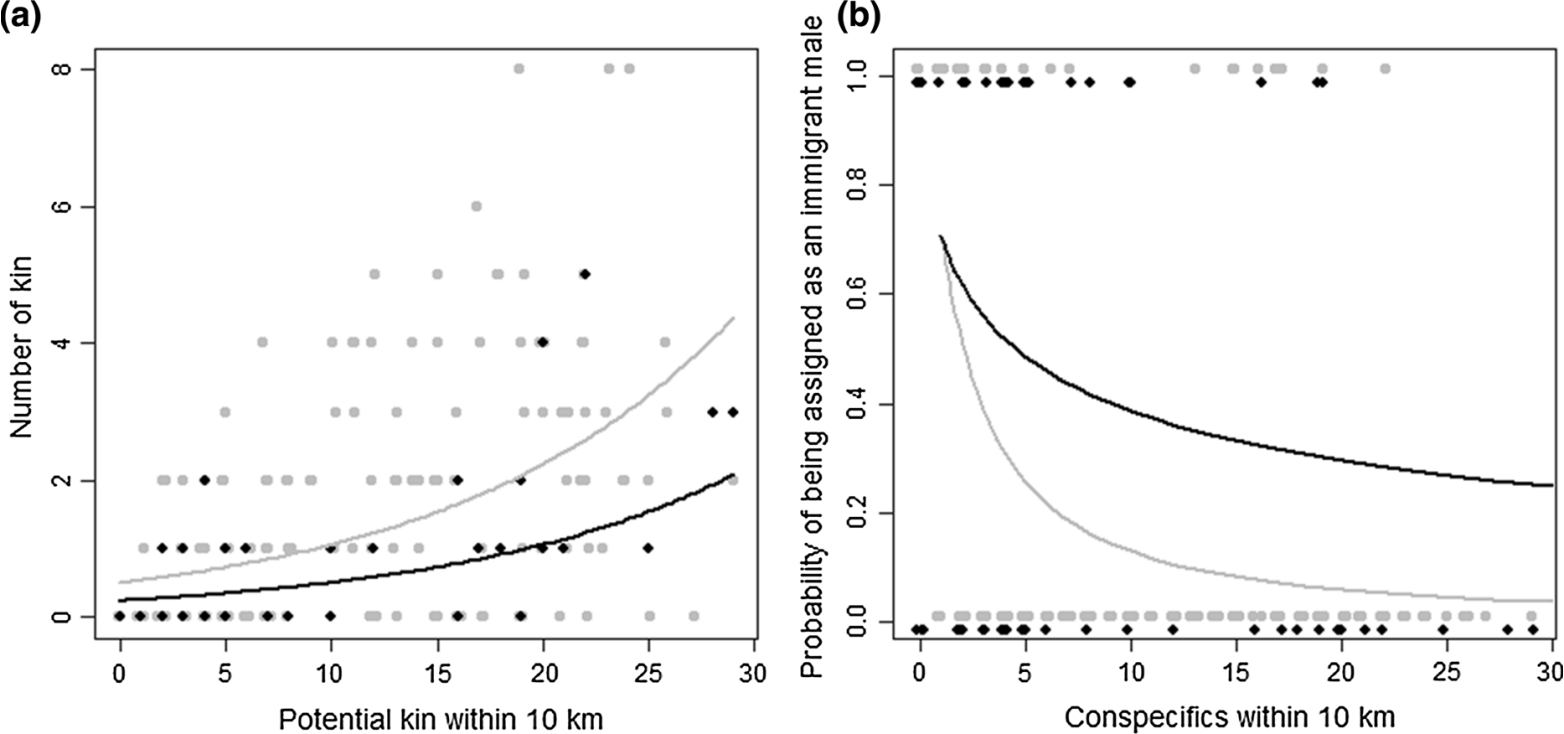
**a** The number of kin assigned to a focal individual and **b** the probability of being assigned as an immigrant male (being male and having no kin within 10 km of location of capture), for individuals captured in areas under the first year (*black points and lines*) and second year (*grey points and lines*) of comprehensive mink control. The *x* axis controls for the number of conspecifics with overlapping life spans (i.e. potential kin) within 10 km of the point of capture of a focal individual

Mink captured in areas in the second year of control had significantly fewer kin (approximately half as many) per neighbouring conspecific than those individuals captured in areas in the first year of control (*p* < 0.0001, Fig. 5a). In the average scenario (i.e. with 10 candidates captured within a 10 km radius of the focal individual), those individuals captured in areas in the first year of control were predicted to have 1.06 kin per 10 conspecifics. In comparison those captured in areas in the second year of control had 1.06 kin per *20* conspecifics. A GLMM directly testing whether the probability of being a male *and* an immigrant (as opposed to any other individual of either sex or inferred dispersal status, and where individuals were classed as immigrants if they had zero kin within a 10 km radius) changed between first and second years of full control (again controlling for the number of conspecifics) showed a strong and significant effect of year of control (*p* = 0.0003). In the average scenario, individuals captured in the second year of control were more than three times as likely to be classed as male immigrants than individuals captured in the first year of control (45 vs 13 %, Fig. 5b).

## Discussion

Overall, this study found evidence of an increased frequency of immigrants following large scale mink control. However, despite apparently long distance dispersal potential, this was not sufficient to overcome the effective rate of decrease through control, combined with sex-biased immigration. We set out to address a number of key questions that would inform our management strategy, whilst also enhancing our understanding of fundamental spatial ecological processes in mammalian mesopredators.

### Compensatory immigration

It is fundamental to the success of an invasive species management project that the rate of removal exceeds the rate of population increase. By examining the relatedness of individuals within 10 km sampling radii, we observed an increase in the frequency of inferred immigrants, with individuals captured in the second year of intensive population control having half as many relatives per conspecific, and an increase from 30 to 50 % of mink that had no relatives at all (i.e. putative immigrants). While such a response is expected from a territorial carnivore such as mink (Gerell 1967; Dunstone 1993), numerically, this was not sufficient to overcome reductions in density of over 60 % between the first and second years of control, and then to a near trivial level by year 3. Moreover, the potential compensatory demographic impact of immigration was diluted by male-biased dispersal. Although there was a 1.7 fold increase in the proportion of putative immigrants, males were observed to disperse further and faster than females, and therefore, as would be expected, the majority of individuals (73 %) captured in the second year of control were male. Modelling the proportion of individuals that were assigned as male immigrants clearly highlighted this, showing a three-fold increase in the proportion of male immigrants between the first and second years of full control.

The confounding effect of dispersal on control efforts was further emphasised by the analysis of changes in mink captured per km of waterway in response to years of control. A subjective discrimination separating mink into two categories simply according to life stage—those that would be expected to be settled, and those that would be expected to be in spatial flux or dispersing—illustrated that whilst control was very effective at rapidly reducing settled mink, the net effect was confounded by dispersing individuals, which were reduced more slowly and less predictably (Fig. 2).

These results provide both an interesting insight into the compensatory immigration potential of mammalian predators in response to culling, as well as the encouraging finding that neither this, nor indeed compensatory fecundity observed in another study (Melero et al. 2015), were sufficient to overcome the impact of large scale coordinated mink control implemented year-round by a network of volunteer citizen conservationists.

### Large scale dispersal

We demonstrated that inferred mink dispersal distances asymptote on average at approximately 38 km for males and 31 km for females, and that these movements frequently connect major river catchments. While our sampling design would have allowed us to detect longer movement, maximum inferred distances were around 55 km for males and 40 km for females. The inferred speed of spread was high, with males commonly moving 15–35 km within the first few months of dispersing (around 240 days in Fig. 3), though this was somewhat lower in females, where the majority had moved <10 km in the same period. Whilst the dispersal kernels obtained from the data give a useful proxy of mink dispersal capacity, it is important to note that they provided a poor fit to the data. This is not surprising, as such descriptive models do not account for probable influential factors such as topography, habitat heterogeneity, the distribution and density of conspecifics, or dispersal behaviour (Zuberogoitia et al. 2013). Nevertheless, the evidence of long distance dispersal implies that protecting species vulnerable to mink predation, such as remnant water vole populations, in a core area requires a large spatial scale for mink control and a buffer exclusion area of at least 30 km radius in order to minimise the potential for seasonal incursions by mink, which may include movement into otherwise suboptimal mink habitat. Even with such an exclusion zone there would still be a requirement for ongoing vigilance, as a small proportion of males (11; 20 %) though fewer females (2; 4 %) may move beyond these distances based on our data, and even a small number of mink can decimate water vole metapopulation networks (Aars et al. 2001). The evidence that dispersal binds mink populations in separate catchments also supports a multi-catchment approach to mink control, as uncontrolled adjacent catchments are within the spatial threshold (i.e. shared watersheds) of being able to provide a supply of immigrants that pose a recolonisation threat. Indeed, individuals from 32 % of pedigree inferred litters were captured in different river catchments, which is consistent with a previous analysis that showed that connectivity (a function of distance to, and number of, mink) to mink in adjacent areas was the strongest factor affecting mink capture rate within a subcatchment (Bryce et al. 2011).

Whilst 3 years of control were sufficient to massively reduce mink density within large river sections, as part of a mainland management area, the CWVCP only gradually achieved systematic coverage of adjacent river sections to eventually form a contiguous management area. Given long distance between-catchment dispersal, it is clear that the asynchronous initiation of mink control in different areas (resulting from local variation in organisational capabilities) must have contributed to recolonisation and delayed the delivery of a large mink-free area. However, even with a large contiguous area bordered by the North Sea on two sides, there remains an ongoing requirement to detect and remove rare re-invading mink, as long as adjacent areas remain uncontrolled, or mink still exist within the control area, albeit at comparatively low densities.

### Heterogeneity in productivity/distribution and connectedness: hotspots

Examining the spatial distribution of female and juvenile mink illustrated a heterogeneous landscape of mink breeding and dispersal with hotspots that accounted disproportionately for juvenile (85 %), adult female (65 %) and immigrant (57 %) mink, relative to the length of waterways (40 %). It could therefore be reasonably assumed that these hotspots represent habitat of high relative quality, and that under optimal habitat selection these areas will be attractive to, and positively selected by, dispersing mink.

The three broad hotspot areas highlighted in Fig. 1 differ markedly in their ecological characteristics, but can all be exploited by the generalist mink. Individually they represented a flood plain of European importance for wetland bird species (the Insh Marshes (Spey catchment): 57°06′09″N, 3°57′14″W), a highly productive lower river and estuarine environment (the Ythan Estuary: 57°20′09″N, 2°00′27″W), and an area of sandy alluvial soils supporting large populations of rabbits, as well as an internationally recognised Atlantic salmon fishery (the River Dee: 57°02′50″N, 2°29′33″W). In a practical sense these areas could be exploited as ‘attractive dispersal sinks’, i.e. areas that are otherwise highly suited for survival and reproduction, and that are positively selected by dispersers, but where demographic rates are in fact negative due to culling (Thomson et al. 1992). Sustained culling of mink dispersing into attractive sinks would effectively reverse the ‘natural’ role of the hotspot areas, where they are likely to make a disproportionately positive contribution to net population growth, and in fact may otherwise act as sources to sink areas, in the classical sense (Holt 1985; Pulliam 1988; Harrison and Taylor 1997). With preferential settlement by dispersers into attractive sinks in below carrying capacity populations, overall population growth rate is expected to drop substantially as the proportion of sink habitat increases. Minimising the growth rate in attractive sinks (e.g. by focussed culling) will reduce the amount of sink habitat required to maintain negative overall population growth rates (Delibes et al. 2001a, b).

Although average mink capture rates approximately halved between the first and second years of full project control, suggesting the current approach to control was effective, this project, in common with many other wildlife management programmes (e.g. Zabala et al. 2010), nevertheless remains dependent on funding packages of fixed duration and is subject to interim lapses in financial support, as well as pressure to deliver more for less resources. Therefore, a management approach with a focus on known hotspot areas, or extrapolating across habitat types, should represent an efficient use of available resources. In particular, such an approach will be more optimal during times when resources are low, when expanding into previously uncontrolled areas, or when reducing the surveillance network following successful population reduction through control. Notwithstanding, it is not yet clear what the consequences of ignoring areas of less suitable habitat may be, particularly if the ultimate goal is eradication.

### Limitations and caveats of the study

Our novel approach of examining changes in population levels and immigration in response to culling efforts through changes in patterns of genetic relatedness, necessitated a number of assumptions and the inference, rather than direct measurement, of the relative contribution of dispersal to local population size. By combining this information with heterogeneity in landscape productivity, changes in sex ratio, and dispersal distances, we were able to gain insights into the large scale spatial ecology of a mammalian predator and how it responds to culling, or increased mortality in general. However, in terms of fully investigating the factors that may be fundamental to compensatory dynamics, we lacked sufficient spatial, or temporal, replication to understand in detail how the net flow of immigrants is affected by spatial variation in habitat quality, density and landscape connectivity (i.e. the permeability of the landscape between points of source and settlement), or whether mink consistently select habitat optimally. A management approach wholly focussed on hotspots would be reliant on immigrants predictably settling in those most productive areas in the landscape.

Our metric of mink ‘density’ should be considered an underestimate as it included the length of all habitat in a river section, some of which may not be suitable for mink. Analysis of long term data, which may identify all habitat actually used by mink would improve estimates of actual density. Notwithstanding, the measurement used here should not be systematically biased and relative changes in the measurement of density used remain valid.

The success in inferring litters by pedigree analysis was variable across the study area being relatively higher in the mid Spey area (the far north-west of the study area, Fig. 1a) where only 18 % of individuals genotyped originated from, but to where 50 % of individuals comprising the inferred litters were assigned. This may reflect that the river catchment, and favourable mink habitat, is more contained by moorland, hence the scope for juveniles dispersing is lower. In contrast, the Cairngorm Mountains (lying to the south east of the Spey) presented something of a barrier. Despite 23 reconstructed litters being present in the Spey, individuals from only 3 of these were connected to individuals captured in river catchments to the east (dispersal distances of 31–36 km). Dispersal seemed to be more fluid across low lying areas. Between the Dee and the Ythan (the east of the study area, Fig. 1a, c) separated by 38–57 km, mink from 6 litters (40 % of trans-catchment litters) were trapped in both catchments, with one additional connection each with the interposed, but lesser controlled area. The individual-level evidence of dispersal was fully consistent with inference based on genetic differentiation of mink sampled across NE Scotland which also revealed the semi-permeable barrier properties of moorland and high elevation portions of the landscape (Zalewski et al. 2009; Fraser et al. 2013). Although this potentially highlights variability in the permeability of the landscape, to address this adequately requires data on settlement (i.e. the breeding territories chosen by immigrant females), and out of the 301 mink for which age, sex and genetic data were available only five qualified as adult (>7 months of age) females that were captured in areas with >1 year of project control (as a proxy for reduced density). Whilst all of these five females were captured in areas we defined as hotspots, a much greater volume of data would be required to investigate how source and settlement habitat quality, and changes in density, affect the dispersal decisions of such individuals, and how this subsequently impacts the spatial dynamics of the system. This remains an outstanding challenge for large-scale spatial ecology and wildlife management research.

A common challenge for invasive species management is that, at least in Europe, funding is short-term and unpredictable. Ensuring a legacy of efforts and impacts in the medium to long term, and despite multiple changes in the level of financial support, requires that resources are targeted effectively. This process can be informed by concentrating resources on those areas that will maximally impact invasive species dynamics, and by understanding dispersal and immigration, which will affect the size of the management area where previous actions can realistically be maintained and defended. This study shows how capture, sex, age and genetic data, collected over 3 years of management interventions, mostly by non-professional volunteer citizen conservationists, provides valuable information to help prioritise the allocation of future resources, both for consolidating the existing project area and expanding into new uncontrolled catchments. We recommend this model for similar eradication initiatives elsewhere particularly those dealing with highly mobile and generalist species.
